# Evaluating the Antidepressant Effect of Verbena officinalis L. (Vervain) Aqueous Extract in Adult Rats

**DOI:** 10.32598/bcn.11.1.3

**Published:** 2020-01-01

**Authors:** Amina Bekara, Ali Amazouz, Tahir Benyamina Douma

**Affiliations:** 1. Laboratory of Natural BioResources, University of Hassiba Benbouali, Chlef, Algeria.

**Keywords:** Aqueous extract, Depression, Neurobehavioral tests, Verbena officinalis L

## Abstract

**Introduction:**

The present study aimed to investigate the antidepressant effect of Verbena (V.) officinalis L. aqueous extract in adult female rats.

**Methods:**

The present study evaluated the antidepressant effect of V. officinalis L. aqueous (V AE) extract in female rats using the Forced-Swimming Test (FST), Light-Dark Box (LDB) test, and Open Field Test (OFT). The level of glycemia and histological analysis were also studied. The VAE [200 mg/kg Parenterally (PO)] was administered orally for 7 successive days in the separate groups of rats.

**Results:**

The oral administration of V. officinalis L. aqueous extract significantly decreased (P< 0.01) the immobility time in the FST, increased the time spent in the light area (LDB), and the number of entry into the central squares (OFT). Thus, the extract at a dose of 200 mg/kg significantly decreased the glycemia level (P<0.05) and suggested no harmful effect on brain histology.

**Conclusion:**

Verbena officinalis L. aqueous extract at a dose of 200 mg/kg could have an anti-depressant effect in adult rats.

## Highlights

- Depression is a highly prevalent disorder in modern society.
- The oral administration of V. officinalis L. aqueous extract significantly decreased the immobility time in the FST.
- The extract at a dose of 200 mg/kg significantly decreased the glycemia level.

## Plain Language Summary

Depression is a highly prevalent disorder in modern society characterized by agitation, insomnia, mood disorders, lack of concentration, and a feeling of guilt. The main causes of depression are hormonal changes and reduction in some neurotransmitters, such as noradrenaline, serotonin, dopamine, and glutamate. This study aimed to investigate the effect of V. officinalis L. leaves aqueous extract on depression in adult female rats using neurobehavioral tests, which can explore depression in animals.our results demonstrated that the brain structure of treated rats with V. officinalis L. aqueous extract was similar to the control group without any injury or hemorrhage. Our results demonstrated that the brain structure of treated rats with V. officinalis L. aqueous extract was similar to the control group without any injury or hemorrhage. The V. officinalis L. aqueous extract at a dose of 200 mg/kg demonstrated an antidepressant effect in an animal model of depression, which was compared to that of fluoxetine.

## 1. Introduction

Depression is a highly prevalent disorder in modern society; it is characterized by agitation, insomnia, mood disorders, lack of concentration, and a feeling of guilt (Santosh et al., 2011). The frequency of depression is higher in women (10%–25%) than men (5%–12%) (Meti et al., 2012). According to the World Health Organization (WHO), depression is a major mental condition affecting nearly 120 million people worldwide. Furthermore, it could become the leading cause of disability in 2020, following cardiovascular diseases.

The main causes of depression are hormonal changes and reduction in some neurotransmitters, such as nor-adrenaline, serotonin, dopamine, and glutamate (Hasler, 2010). A wide range of drugs are applied to treat depression, such as like tricyclics, the selective, reversible inhibitors of monoamine oxidase, selective serotonin reup-take inhibitors, and specific serotonin–noradrenaline reuptake inhibitors, but they are insufficient (Richelson, 1994; Taylor & Stein, 2005). Anti-depressant drugs have several adverse effects, such as intolerance, dizziness, dependence, and so on.

Phytotherapy may substitute these chemical molecules. This is because it has been considered as relatively safe; furthermore, the search of a new herbal source of therapy has progressed seriously (Zhang, 2004). The most common antidepressant plants are Lavandula officinalis, Zingiber officinal, Centella asiatica, Hypericum perforatum, Withania somnifera, and Verbena officinalis L (Khare, 2007).

Verbena officinalis L (vervain) belongs to the family of Verbenaceae and grows all around the world, especially in Europe and Asia (Shu, Liu, & Chou 2012). This plant has been investigated for its anti-depressant (Jawaid, Kamal, & Andimam, 2015), anti-inflammatory (Calvo, 2006), anti-bacterial (Hernandez, Tereschuk, & Abdala, 2000), and anti-tumor (Kou et al., 2013) effects.

Besides, the Vervain extract is highly rich in bioactive compounds, such as verbenin, ursolic acid, monoterpenes, and terpenoids (Rehecho et al., 2011). Several studies have reported that tannins and flavonoids in Ver-vain extracts have anxiolytic and antidepressant activities in the experimental model. In addition, this plant has shown a sedative effect that acts especially on the central nervous system (Adeyemi, Yemitan, & Taiwo, 2006; Coleta et al., 2008; Aguirre-Hernandez et al., 2010). The work on the antidepressant effect of V. officinalis L. is recent and in small numbers, leaving this area open for further studies.Therefore, the present study aimed to investigate the effect of V. officinalis L. leaves aqueous extract on depression in adult female rats using neurobehavioral tests, which can explore depression in animals.

## 2. Methods

The V. officinalis L. dried leaves were obtained from a local herb market in Chlef center (Algeria), in January 2018. A voucher specimen was deposited in the herbarium of the Chlef University for future reference.

Dried leaves were grounded, then, 25 g of the obtained powder was macerated in hot water (100°C) for 15 minutes to achieve the plant infusion. Next, the extract was filtered and lyophilized to obtain a brownish powder with a yield of 15, 54% (W/V) (Hosseinzadeh, Tafaghodi, Abedzadeh, & Taghiabad, 2013).

The obtained extract of V. officinalis L. was investigated for the presence or absence of some bioactive compounds, such as polyphenols, flavonoids, alkaloids, saponins (Sofowora, 1993) tannins (Trease & Evans 2002), resins and terpenoids (Aiyegoro & Okoh, 2010).

Adult female rats (N=164, weight: 89±13 g) were obtained from the Pasteur Institute (Algeria). The animals were maintained in the animal house of Natural Biore-sources laboratory, Department of Biology, Nature and Life Sciences Faculty, University of Hassiba Benbouali, Chlef (Algeria). The study rats had access to water and standard diet, ad libitum.

Fluoxetine (20 mg/kg) was used as a reference standard for the anti-depressant activity. The animals were divided into three groups, each consisting of 7 rats as following (Jawaid et al., 2015), the extract was orally administered to the rats by gavage in the morning (Table 1).

**Table 1.** Experimental groups

**Group**	**??**
I: Control	Normal saline solution 1 mL b.w orally/7 days
II : Standard	Fluoxetine 20 mg/kg b.w orally/7 days
III: Test drug	V. officinalis L. aqueous extract (V.O.A) at a dose of 200 mg/kg b.w orally/7 days

b.w: Body Weight; n=7

After 24 hours of the last treatment, all study rats were exposed to the neurobehavioral tests. The Forced Swimming Test (FST) is the most widely used in vivo tests to determine the antidepressant activity of molecules. The apparatus is a glass cylinder (40 cm × 20 cm) filled with water (25°C). The animal is placed into the cylinder, and the time of immobility is recorded from a total testing time of 6 minutes. The development of immobility time by the animal is considered as a good indicator of depressive behavior; this is because the cessation of swimming reflects the animal’s despair from escaping this situation (Porsolt et al., 1977).

The Light-Dark Box (LDB) test constitutes a wooden box (44 cm × 21 cm × 21 cm) divided into 02 compartments of light and dark. Rats usually avoid the light areas; however, they have a significant capacity to explore a new compartment. This situation creates conflict; therefore, the more the animal is anxious, the more it spends time in the dark area. The recorded parameter is the time (seconds) spent in the light area.

The apparatus is made of wood (50 cm × 25 cm × 50 cm) and is divided into 16 equal squares. In general, animals extremely avoid the central area, compared to the periphery; thus, antidepressant molecules increase the number of entries to the center (Walsh and Cumminus, 1976).

After conducting behavioral tests, the study rats were sacrificed in the morning; blood was collected from portal vein in tubes, then used to determine the blood glucose level by a glucometer (Kahloula et al., 2013).

The required samples were taken from the study rats’ brains and fixed in formalin (10%). Then, they were washed under tap water and introduced in a bath containing the serial dilutions of graduated alcohol (methyl, ethyl, & absolute ethyl). They were then used for dehydration. Accordingly, the same samples were cleared in xylene and embedded in liquid paraffin at 56°C. Next, the sections of 4 μm of thickness were cut, deparaffinized, and stained with hematoxylin/Eosin stains for histopathological examination under a light microscope (40×100).

All obtained results were expressed as mean±SEM (Standard of Error). The Kruskal Wallis test and Dunn’s posthoc test were used to examining the significance level between the study groups. P<0. 05 was considered as statistically significant. The data analysis was performed using statistical software R (Team, 2010; R.D.C: a language and environment for statistical computing, Vienna, Austria; R: foundation for statistical computing retried from http/www.R.project.org).

## 3. Results

The phytochemical screening revealed that the aqueous extract of V. officinalis L. is rich in polyphenols, flavonoids, tannins, and terpenoids (Table 2). However, we noted the absence of other compounds, such as saponins, resins, alkaloids, and steroids. These results were obtained after several reactions of coloration and precipitation, and based on the observations, we recorded the presence or the absence of each compound (Table 2).

**Table 2.** The phytochemical screening results of V. officinalis L. aqueous extract

**Compound**	**Result**
Polyphenols	+++
Flavonoids	+++
Tannins	++
Alkaloids	−
Resins	−
Saponins	−
Terpenoides	++
Steroids	+

^+++^ Highly present; ^++^Moderately present; ^−^ Absence

**Figure 1.**
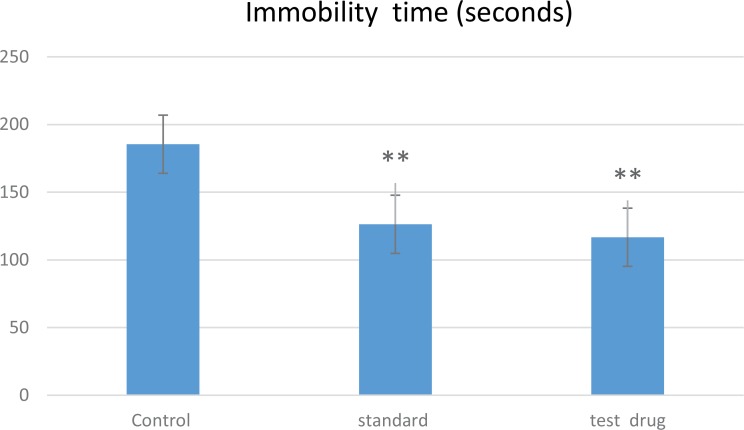
FST data in terms of the time of immobility in seconds A comparison was conducted between the control vs. standard and standard vs. test drug groups. Th control (rats treated with saline solution), standard (rats treated with 20 mg/kg of fluoxetine), and test drug (rats treated with 200mg/kg of V. officinalis L. aqueous extract/7 days) groups. Values are expressed in mean±STE (n=7) ^**^P<0.001.

**Figure 2.**
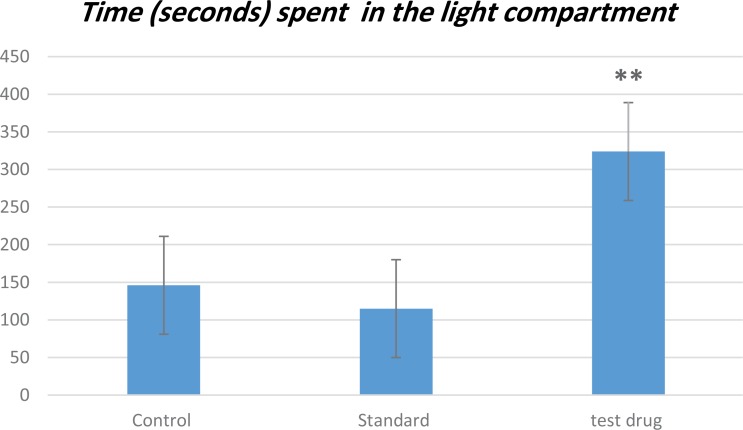
FST The results of LDB test in terms of time (in seconds) spent in the light compartment by the rats of all groups A comparison was conducted between the control vs. standard and standard vs. test drug groups. The controls (rats treated with saline solution), standard group (rats treated with 20 mg/kg of fluoxetine), and test drug group (rats treated with 200mg/kg of V. officinalis L. aqueous extract/7 days). Values are expressed in Mean±STE (n= 7) ^**^P<0.01.

In this test (Figure 1), the study rats treated by 200mg/kg of V. officinalis L. aqueous extract for one week indicated a significant decrease in immobility time (116.72±8.38 seconds), in comparison with the control group that received saline solution (185.42±17.31 seconds), and the standard group that received 20 mg/kg of fluoxetine (126.28±7.66 seconds), respectively (P<0.01). The result of the LDB test is represented in Figure 2. The treatment of rats by the aqueous extract of V. officinalis L. at dose of 200 mg/Kg for 7 successive days significantly increased (P<0.01) the time spent in the light compartment (323.85±34 seconds), compared to the other groups; control (146±31.87 seconds) and standard (115± 37.30 seconds) groups.

The effect of oral administration of V. officinalis L. aqueous extract on rats’ behavior is presented in Table 3. For a dose of 200 mg/kg of plant, we recorded a non-significant augmentation of the number of visits in the central squares of treated rats (7±1.87), compared to other groups; control (2.14±0.73) and standard (2.85±1.45) groups, respectively.

**Table 3.** The results of the Open Field Test

**Groups**	**Number of Entries in the Central Squares (Mean±STE)**
Control (saline solution)	2.14±0.73
Standard (fluoxetine 20 mg/kg)	2.85±1.45
Test drug (200 mg/kg of V. officinalis L aqueous extract)	7±1.87

A comparison was performed between the control vs. standard and standard vs. test drug groups. Values are expressed in Mean±STE (n=7);

** P<0.01

**Figure 3.**
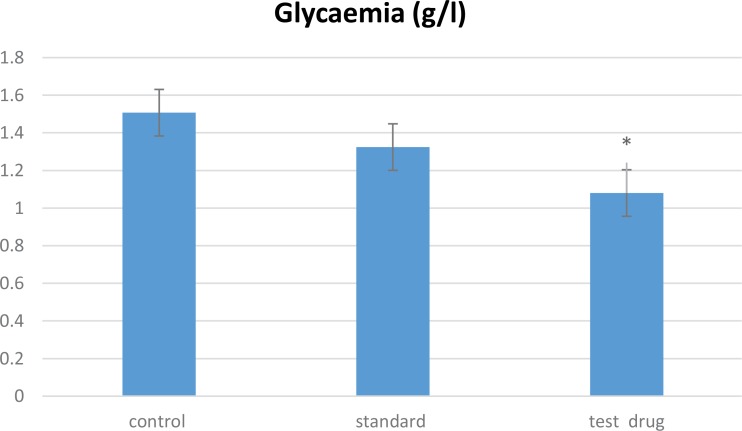
The results of the dose of glycemia (g/L) of all study rats A comparison was conducted between the control vs. standard and standard vs. test drug groups. The control (rats treated with saline solution), standard (rats treated with 20 mg/kg of fluoxetine), and test drug (rats treated with 200mg/kg of V. officinalis L. aqueous extract/7 days) groups. Values are expressed in Mean±STD (n= 7); P<0.05.

The result of glycemia test is shown in Figure 3. We observed that the oral administration of V. officinalis L. aqueous extract (200 mg/kg for 7 days) significantly decreased (P<0.05) the glycemia in the test group (1.19±0.06 g/L), in comparison with other groups; control (1.5±0.1 g/L) and standard (1.32±0.08 g/L) groups, respectively.

**Figure 4.**
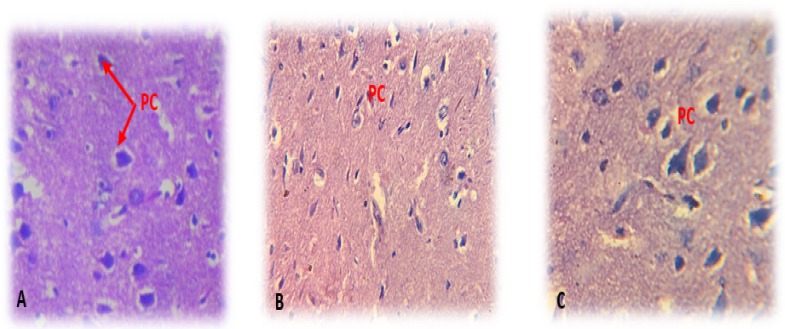
The histological sections of rats’ brain stained by hematoxylin-eosin and observed under a light microscope (40×10) A: Control group treated with saline solution, B: Standard group treated with Fluoxetine 20 mg/kg and C: Test drug group treated with 200mg/kg of V. officinalis L. aqueous extract. PC: Pyramidal cells. (40×100).

The histological examination data are represented in Figure 4. Treating the study rats with 200 mg/kg of V. officinalis L. aqueous extract for 7 successive days had no harmful effect on the brain histology of tested animals, which was similar to the controls (saline solution) and the standard group (fluoxetine 20 mg/kg). The brain histology of all rats was well arranged with normal pyramidal cells in density and size; besides, we noted a total absence of hemorrhage or lesion.

## 4. Discussion

Depression is a highly prevalent disorder in the modern society; therefore, phytotherapy may be an effective alternative treatment for this disorder to avoid excessive use of drugs, which have several adverse effects (Harsat et al., 1997).

In this regard, the oral administration of V. officinalis L. aqueous extract (vervain) at a dose of 200 mg/kg indicated a significant decrease in the immobility time in the FST. This result is per the study of Jawaid et al. (2015). The diminution of the immobility time may be attributed to the terpenes that can modulate the dopaminergic system involved in the physiopathology of depression (Machado et al., 2012). Moreover, we recorded a significant increase in the time spent in the light compartment of the LDB test after treating the study rats with plant extract. This enhancement was mainly due to the presence of bioactive compounds, such as flavonoids and tannins that contributed to the anxiolytic effect of Verbena (Mengiste, Yesuf, & Getachew, 2014; Edwards et al., 2015). Our results were in line with those of Khan, Khan, & Ahmed, (2016).

Besides, in the OFT, we noted that the oral administration of vervain extract increased the number of visits in the central squares; this augmentation is considered a good indication of the anxiolytic effect. Our results were consistent with those of Khan et al. (2016).

However, the oral treatment of rats with vervain extract at dose of 200 mg/kg significantly decreased the glycemia; this effect may be explained by the presence of bioactive compounds in the plant extract, like triterpenes which have an antidiabetic activity according to previous studies (Alqahtani et al., 2013, Rehecho et al., 2011).

For the histological study, our results demonstrated that the brain structure of treated rats with V. officinalis L. aqueous extract was similar to the control group without any injury or hemorrhage. Our findings were in line with prior studies (Rashidian et al., 2017).

The aqueous extract of V. officinalis L. contains several bioactive compounds, such as polyphenols, flavonoids, tannins, and terpenoids. Our results were consistent with the previous works, which suggested that the beneficial effect of this plant is attributed to the presence of these constituents in the extract (Calvo et al., 1997; Siddiqui & Verma, 2001; Bilia et al., 2008; Rehecho et al., 2011).

The precise mechanisms of the antidepressant effect of V. officinalis L. aqueous extract are not completely understood; thus, further studies are necessary to determine which compounds are responsible for these effects.

## 5. Conclusion

The oral administration of V. officinalis L. aqueous extract at a dose of 200 mg/kg demonstrated an antidepressant effect in an animal model of depression, which was compared to that of fluoxetine (20 mg/kg) in this study. The phytochemical screening revealed the presence of polyphenols, flavonoids, tannins, terpenoids, and steroids that contributed to the observed effect.
